# Effect of a single dose of oral azithromycin on malaria parasitaemia in children: a randomized controlled trial

**DOI:** 10.1186/s12936-021-03895-9

**Published:** 2021-08-31

**Authors:** Boubacar Coulibaly, Ali Sié, Clarisse Dah, Mamadou Bountogo, Mamadou Ouattara, Adama Compaoré, Moustapha Nikiema, Jérôme Nankoné Tiansi, Nestor Dembélé Sibiri, Jessica M. Brogdon, Elodie Lebas, Thuy Doan, Travis C. Porco, Thomas M. Lietman, Catherine E. Oldenburg

**Affiliations:** 1 Centre de Recherche en Santé de Nouna, Nouna, Burkina Faso; 2 Francis I Proctor Foundation, University of California, San Francisco, 490 Illinois St, Floor 2, San Francisco, CA 94143 USA; 3 Department of Ophthalmology, University of California, San Francisco, San Francisco, CA USA; 4 Department of Epidemiology and Biostatistics, University of California, San Francisco, San Francisco, CA USA

**Keywords:** Malaria, Azithromycin, Burkina Faso, Randomized controlled trial

## Abstract

**Background:**

Azithromycin has recently been shown to reduce all-cause childhood mortality in sub-Saharan Africa. One potential mechanism of this effect is via the anti-malarial effect of azithromycin, which may help treat or prevent malaria infection. This study evaluated short- and longer-term effects of azithromycin on malaria outcomes in children.

**Methods:**

Children aged 8 days to 59 months were randomized in a 1:1 fashion to a single oral dose of azithromycin (20 mg/kg) or matching placebo. Children were evaluated for malaria via thin and thick smear and rapid diagnostic test (for those with tympanic temperature ≥ 37.5 °C) at baseline and 14 days and 6 months after treatment. Malaria outcomes in children receiving azithromycin versus placebo were compared at each follow-up timepoint separately.

**Results:**

Of 450 children enrolled, 230 were randomized to azithromycin and 220 to placebo. Children were a median of 26 months and 51% were female, and 17% were positive for malaria parasitaemia at baseline. There was no evidence of a difference in malaria parasitaemia at 14 days or 6 months after treatment. In the azithromycin arm, 20% of children were positive for parasitaemia at 14 days compared to 17% in the placebo arm (*P* = 0.43) and 7.6% vs. 5.6% in the azithromycin compared to placebo arms at 6 months (*P* = 0.47).

**Conclusions:**

Azithromycin did not affect malaria outcomes in this study, possibly due to the individually randomized nature of the trial.

*Trial registration* This study is registered at clinicaltrials.gov (NCT03676751; registered 19 September 2018).

**Supplementary Information:**

The online version contains supplementary material available at 10.1186/s12936-021-03895-9.

## Background

Biannual mass azithromycin distribution with a single oral 20 mg/kg dose has been shown to reduce all-cause childhood mortality in settings with high malaria transmission [1]. In this same study, azithromycin was shown to lead to a decrease in malaria-specific mortality and in malaria parasitaemia at the community level [2, 3]. In other high malaria settings, increased frequency of azithromycin distribution for trachoma control may reduce malaria transmission during the low transmission season [4, 5], although findings are mixed in other studies [6, 7]. Azithromycin has activity against the plasmodial apicoplast and may reduce transmission in communities or help treat or prevent infections in conjunction with other anti-malarials [8–11]. Azithromycin has also been considered in combination with anti-malarials for prevention and treatment of malaria in pregnancy and in children, with results typically showing that it is well tolerated and safe in pregnancy, but not superior to sulfadoxine–pyrimethamine or artemether–lumefantrine [12–15].

Prevention or treatment of existing malaria infection may be one mechanism of any effect of single dose oral azithromycin on all-cause childhood mortality, particularly where malaria is an important cause of child mortality. This individually randomized trial of single oral azithromycin compared to matching placebo was designed to elucidate potential mechanisms of any effect of azithromycin on mortality in Nouna, Burkina Faso. A pre-specified secondary analysis of the trial evaluated the effect of single dose azithromycin on malaria parasitaemia administered at the end of the high malaria transmission season in a region with predominantly *Plasmodium falciparum* malaria.

## Methods

### Study overview

The GAMIN study was an individually randomized 1:1 trial comparing a single oral dose of azithromycin (20 mg/kg) or matching placebo to better understand potential mechanisms for reducing child mortality in children aged 0–59 months in Burkina Faso. Assessments were performed at baseline, 14 days from enrollment, and 6 months from enrollment. The study was reviewed and approved by the Institutional Review Board at the University of California, San Francisco and the Comité National d’Ethique pour la Recherche (National Ethics Committee of Burkina Faso) in Ouagadougou, Burkina Faso. Written informed consent was obtained from the caregiver of each enrolled participant.

### Study setting

Participants were enrolled in Nouna Town, Burkina Faso. Nouna is the capital of Kossi Province and located in the northwest of Burkina Faso, with a population of approximately 20,000 residents. As with much of the Sahel, Nouna has highly seasonal malaria transmission, with transmission peaking during the rainy season from approximately July through October [16]. Children were enrolled in November 2019, at the end of the rainy season, and the final follow-up visit was in June 2020, prior to the beginning of the rainy season. Thus, all children were enrolled at the end of the high malaria transmission season and followed during the low transmission season.

### Recruitment and eligibility

Nouna Town is located in the Nouna Health and Demographic Surveillance Site (HDSS) [17], which conducts regular census of all participating communities. Study staff visited households with children under 5 in residence based on the census and in consultation with key informants living in the communities. Caregivers were informed about the study, and if interested, asked to bring their child to the Nouna District Hospital for eligibility assessment and enrollment. Children were eligible if they: (1) were between 8 days and 59 months of age; (2) were resident of the town of Nouna and not planning on moving in the next 6 months; (3) were able to feed orally (to take the study medication); and (4) had no known allergies to macrolides.

### Intervention

Azithromycin and matching placebo were prepared as pediatric oral suspension (both provided by Pfizer Inc, New York, NY). A single oral 20 mg/kg dose was offered to each participant or an equivalent volume of placebo. Dosing was weight-based for children who were too young to stand, and a height stick approximation was used for children over 12 months of age, as in trachoma control programmes and has been previously done in studies of azithromycin for prevention of childhood mortality (Additional file 2: Fig. S1) [18, 19]. All study treatments were directly observed, and study staff recorded on the electronic study application whether or not the child received their medication dose.

### Randomization and masking

Children were randomized in a 1:1 fashion to azithromycin or matching placebo. The randomization sequence was generated without blocking or stratification by TCP and WWG. Study identification numbers were tied to randomization assignments in the study’s electronic data capture system. Allocation concealment was achieved as the electronic data capture system did not reveal the randomized treatment assignment until after the eligibility assessment, enrollment, and baseline assessment were complete. The placebo preparation was identical in contents to azithromycin with the exception of the active ingredient. Azithromycin and placebo were identical in taste, smell, and appearance, and labels were identical with the exception of a random letter used to identify contents of the bottle. All members of the study team with the exception of the study biostatisticians were masked to which letters identified azithromycin and placebo. Participants were not aware of whether they received azithromycin or placebo.

### Malaria assessment

At each study visit (baseline, 14 days, and 6 months), thin and thick smears were collected from each study participant for assessment of malaria parasitaemia. Blood smears were collected on glass slides, air dried, and stored at room temperature. Smears were stained with 3% Giemsa and read by two masked microscopists who determined the presence or absence of *Plasmodium* parasites, number of asexual parasites per µl (counting 200 white blood cells and assuming 8000 white blood cells per µl) to assess parasite density, and presence of gametocytes. Discrepancies were adjudicated by a third masked microscopist. A positive smear was considered positive for malaria. The median of the two parasite density measurements was used for analysis. Tympanic temperature was measured in all children at all time points. Those with fever (defined as temperature ≥ 37.5 °C) were administered a rapid diagnostic test (RDT) for malaria. Children with a positive malaria RDT were referred for appropriate care.

### Sample size

The sample size was determined based on the primary outcome of the trial, which was Shannon’s diversity index of the gut microbiome. A sample size of 225 children per arm (450 total) would provide at least 80% power for a detectable effect size of 1.02 standard deviations of Shannon’s diversity with no loss to follow-up. Under this fixed design, 225 children per arm would have approximately 80% power to detect an 11% absolute difference in malaria parasitaemia, assuming 18.5% prevalence in the placebo arm at 14 days.

### Statistical methods

Baseline demographic variables were summarized with medians and interquartile ranges (IQR) for continuous variables and proportions for categorical variables. The prevalence of parasitaemia, gametocytaemia, and parasitaemia plus fever were calculated at each time point with binomial 95% confidence intervals. Parasite density measurements were log transformed for analysis. Analyses of parasite density were restricted only to those who had positive malaria smears. Unadjusted logistic regression models to estimate odds ratios (OR) and 95% confidence intervals (CI) were used to estimate the effect of azithromycin versus placebo on each dichotomous malaria outcome, and an unadjusted linear regression model to estimate mean differences in parasite density in children receiving azithromycin compared to placebo. As a non-pre-specified subgroup analysis, 14-day malaria parasitaemia models were stratified by malaria parasitaemia positivity at baseline (presence or absence of parasites). Per the study’s pre-specified analysis plan, all analyses were intention-to-treat and children were analysed as per their random treatment assignment. As a sensitivity analysis, children who did not receive their random treatment assignment were excluded, following the same analyses outlined for the intention-to-treat analysis. For parasite density outcomes at 14 days, a sensitivity analysis adjusting for baseline parasite density was conducted. *P* < 0.05 was considered statistically significant. No adjustments were made for multiple comparisons. All analyses were conducted in Stata version 15.1 (StataCorp, College Station, TX).

## Results

**Fig. 1:**
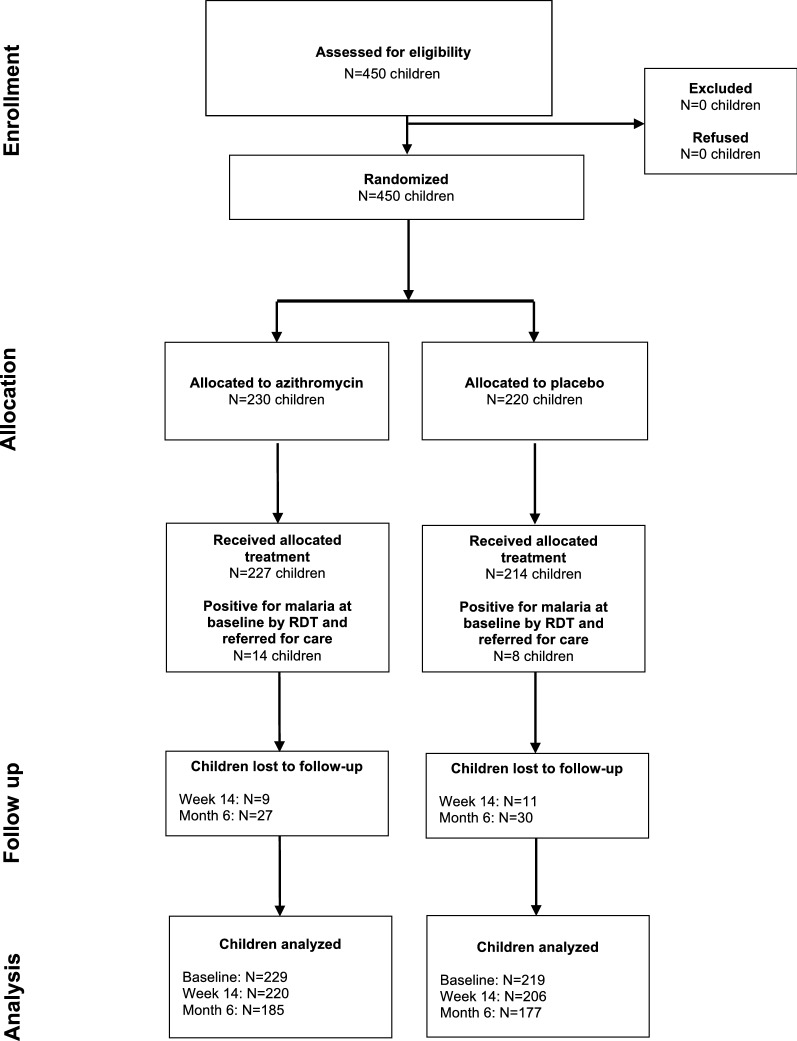
CONSORT flow diagram of study participants

Of 450 children screened, all were eligible and enrolled in the trial, and 230 were randomized to azithromycin and 220 to placebo (Fig. 1). Three (1.3%) children in the azithromycin arm and 6 (2.7%) children in the placebo arm did not receive their allocated treatment assignment because they vomited or spit up after treatment administration. Children in both treatment groups were a median of 26 months old, and 53 and 48% were female in the azithromycin and placebo groups respectively (Table 1). Malaria parasitaemia was balanced between treatment groups at baseline prior to study treatment and was present in 18 and 16% of children in the azithromycin and placebo groups. Adverse events at 14 days following treatment administration were similar between treatment groups as previously described [20].

**Table 1:** Baseline characteristics by treatment arm

	Azithromycin (N = 230)	Placebo (N = 220)
Age, months, median (IQR)	26 (15 to 39)	26 (17 to 38)
Female sex, N (%)	122 (53.1%)	106 (48.4%)
Currently breastfeeding, N (%)	93 (40.4%)	78 (35.6%)
Mother’s age, years, median (IQR)	26.5 (23 to 32)	27 (23 to 33)
Malaria parasitaemia, N (%)	42 (18.3%)	36 (16.4%)
Parasite density, parasites/µl, median (IQR)	960 (84 to 15,900)	1160 (80 to 9940)
Gametocytaemia, N (%)	37 (16.2%)	26 (11.9%)
Positive RDT for malaria^a^	14 (6.1%)	8 (3.6%)

^a^Rapid diagnostic test (RDT) for malaria was performed in children with tympanic temperature ≥ 37.5 °C; children with a positive RDT at baseline were referred for anti-malarial care

**Fig. 2:**
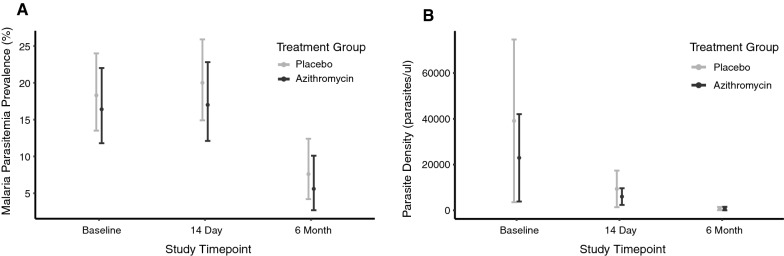
Malaria parasitaemia prevalence (**A**) and parasite density in parasites/µl (restricted only to those with a positive smear for parasitaemia) (**B**) at baseline, 14 days, and 6 months by treatment arm. Black lines indicate azithromycin and gray lines indicate placebo

No evidence of a difference between the azithromycin and placebo groups for any malaria outcomes were observed (Table 2). Malaria parasitaemia declined from baseline to 6 months in similarly in both study arms (Fig. 2), as expected since the 6-month visit was conducted in June prior to the beginning of the high malaria transmission season. At 14 days, 20% of smears in the azithromycin arm were positive for parasites compared to 17% in the placebo arm (OR 1.22 for azithromycin vs. placebo, 95% CI 0.75 to 2.00, *P* = 0.43). Restricting models only to children who received their allocated study treatment did not affect results for any of the outcomes (Additional file 1: Table S1). At 14 days, the mean difference in log parasite density was similar in unadjusted models (mean difference − 0.44, 95% CI − 1.60 to 0.71, *P* = 0.45) and adjusted for baseline parasite density (mean difference − 0.76, 95% CI − 2.67 to 1.16, *P* = 0.43).

**Table 2:** Malaria outcomes by treatment group

	Azithromycin N (%) or median (IQR)	Placebo N (%) or median (IQR)	Odds ratio or mean difference (95% CI)	*P*-value
Malaria parasitaemia				
14 days	44 (20.0%)	35 (17.0%)	1.22 (0.75 to 2.00)	0.43
6 months	14 (7.6%)	10 (5.6%)	1.37 (0.59 to 3.16)	0.47
Parasite density^a^				
14 days	188 (60 to 7087.5)	520 (120 to 5800)	− 0.44 (− 1.60 to 0.71)	0.45
6 months	108 (64 to 540)	256 (88 to 790)	− 0.21 (− 1.55 to 1.12)	0.74
Gametocytaemia				
14 days	39 (17.7%)	31 (15.1%)	1.22 (0.73 to 2.04)	0.46
6 months	14 (7.6%)	9 (5.1%)	1.53 (0.64 to 3.63)	0.34
Parasitaemia plus fever^b^				
14 days	7 (3.2%)	4 (1.9%)	1.66 (0.48 to 5.75)	0.43
6 months	3 (1.6%)	1 (0.6%)	2.90 (0.30 to 28.2)	0.36

*IQR* interquartile range *CI* confidence interval

^a^Among children with a positive smear, with models using a log transformation of parasite density

^b^Fever defined as tympanic temperature ≥ 37.5 °C

In stratified models, there was no evidence of a difference in malaria parasitaemia prevalence at 14 days in children who were negative for malaria parasitaemia at baseline (OR 1.11 for azithromycin vs. placebo, 95% CI 0.57 to 2.17, *P* = 0.75) nor in children who were malaria parasitaemia positive at baseline (OR 1.69 for azithromycin vs. placebo, 95% CI 0.68 to 4.19, *P* = 0.26; *P*-value for interaction for treatment group by baseline malaria status = 0.47).

## Discussion

No evidence that a single dose of oral azithromycin reduces malaria infection was observed in this community-based sample of children in a setting in Burkina Faso with highly seasonal malaria transmission. A household randomized trial of azithromycin versus placebo given alongside seasonal malaria chemoprevention for 3 consecutive years of administration in children aged 2 to 59 months showed no additional effect of azithromycin on malaria parasitaemia in Burkina Faso and Mali [21]. However, secondary analysis from this trial found a short-term reduction in rapid diagnostic test-confirmed malaria in children receiving azithromycin compared to placebo, confined to approximately 14 days post-treatment [22]. A study of biannual mass azithromycin distribution in Niger among children aged 1 to 59 months old found a nearly 50% reduction in the odds of malaria parasitaemia in children living in communities receiving azithromycin compared to placebo [3]. In that study, malaria outcomes were measured during the low transmission season (approximately March–April), with study treatment distributions occurring biannually in approximately July (high transmission season) and January (low transmission season). Communities in the Niger study were not receiving seasonal malaria chemoprevention. One potential explanation for differences in the findings of the two studies is that azithromycin may confer no additional effect on malaria transmission in the presence of seasonal malaria chemoprevention, which has strong effects on malaria parasitaemia. Although the study area for the present study receives 4 monthly distributions of seasonal malaria chemoprevention from July through October, the study was conducted after the annual seasonal malaria chemoprevention campaign had ended. Study treatments were administered approximately 1 month after the last round of seasonal malaria chemoprevention.

One possible explanation for discordant results between this study, which found no effect of azithromycin at 14 days or 6 months, and previous studies showing short- and longer-term effects of malaria may be the proportion of the population receiving azithromycin. Mass drug administration may be more effective for control of infectious disease than individual-level treatment because it may disrupt transmission in communities. In the Niger study of biannual mass azithromycin distribution, all preschool children in an entire community were treated on the same day and may have introduced some herd-like protection of untreated individuals by reducing community transmission [3]. The parent trial for this study also found a reduction in malaria mortality following biannual mass azithromycin distribution [2]. In the household-randomized study in Burkina Faso and Mali, some transmission may have been reduced by treatment at the household level, explaining short-term reductions in malaria positivity, but since entire communities were not treated at the same time, this may not have conferred community-level transmission reduction.

There was no evidence of a difference in 14-day malaria results in models stratified by baseline malaria status, although the sample size was small, especially for models restricted only to those with positive baseline smears. Azithromycin has weak anti-malarial properties and is not typically considered suitable as monotherapy for treatment of malaria [23]. Studies of azithromycin specifically for malaria (rather than childhood mortality or trachoma) have typically evaluated azithromycin plus an anti-malarial such as chloroquine. It is possible that effects seen in previous studies including azithromycin are due to the anti-malarial rather than azithromycin itself. Children with a positive RDT were referred for care, which includes receipt of anti-malarials. However, this likely did not substantively affect results as very few children were RDT positive. The present study evaluated a single 20 mg/kg dose, consistent with what has been used in trachoma control programmes and studies of azithromycin for prevention of childhood mortality. A higher dose or repeat dosing may have more effect against malaria. For example, in a study of azithromycin plus chloroquine in adults in India, Colombia, and Suriname, a 1 g dose (equivalent to that used for adults in trachoma programmes) in combination with chloroquine was inferior to sulfadoxine–pyrimethamine plus chloroquine, but response rates improved with a 2 g dose of azithromycin [24]. However, the results of the present trial do not provide evidence of a preventative benefit for malaria infection in young children in peri-urban Burkina Faso with the dose currently being considered for implementation for prevention of childhood mortality.

The results of this study must be considered in the context of several limitations. This trial was designed around the primary endpoint of evaluating the paediatric microbiome, and thus was not specifically powered for malaria outcomes. This limited statistical power especially in subgroup analyses by baseline malaria status. Although there was no significant effect of azithromycin on any malaria outcome, point estimates for some models suggested that children receiving azithromycin had worse malaria outcomes than those receiving placebo. This may have been due to a chance imbalance in malaria parasitaemia at baseline. Children with a baseline infection may have been at higher risk of repeated infection, for example, due to environmental or behavioural factors such as improved housing materials or proximity to standing water, or children with positive smears at baseline may have still been positive at 14 days [25, 26]. Models restricted to negative baseline malaria parasitaemia were closer to the null than the main models, suggesting that there is likely no true effect of azithromycin on malaria outcomes in this study. While the dose may have been too low to achieve a therapeutic effect, this dose is currently under consideration for implementation for prevention of childhood mortality and its evaluation is useful for better elucidating potential mechanisms of the effect of azithromycin on childhood mortality. Finally, this study was conducted in a peri-urban area of Burkina Faso during the low transmission season for malaria. The proportion of children who were positive for malaria by smear was lower in June than November/December, in line with malaria epidemiology in the region. Because malaria transmission patterns may be different in more rural settings and during higher transmission periods, these results may not be generalizable to different geographic locations or to the rainy season.

## Conclusions

In this individually randomized trial, there was no evidence of an effect of a single oral dose of azithromycin on malaria outcomes in children under 5 years of age. Individual-level treatment of children with azithromycin with a single dose may not be sufficient to affect malaria transmission and thus outcomes compared to community distributions.

## Supplementary Information

**Additional file 1: Table S1.** Malaria outcomes by treatment group among children who received their allocated study treatment.

**Additional file 2.** Height stick for height-based dosing for children aged 12-59 months, Burkina Faso.

## Data Availability

Data underlying the above analyses are available upon reasonable request from the corresponding author.

## Abbreviations

CI: Confidence interval
HDSS: Health and Demographic Surveillance Site
IQR: Interquartile range
OR: Odds ratio
