# Supplementary material for: Effect of a single dose of oral azithromycin on malaria parasitaemia in children: a randomized controlled trial
Source: Malar J. 2021 Aug 31;20:360. doi: 10.1186/s12936-021-03895-9 (PMC8407066; doi:10.1186/s12936-021-03895-9)
Supplement: Supplementary file 1 — Additional file 1: Table S1. Malaria outcomes by treatment group among children who received their allocated study treatment. [file 12936_2021_3895_MOESM1_ESM.docx]

**Table S1.** Malaria outcomes by treatment group among children who received their allocated study treatment

|  | **Azithromycin**  **N (%) or**  **Median (IQR)** | **Placebo**  **N (%) or**  **Median (IQR)** | **Odds Ratio or Mean Difference**  **(95% CI)** | ***P*-value** |
| --- | --- | --- | --- | --- |
| Malaria parasitemia |  |  |  |  |
| 14 days | 42 (19.4%) | 32 (16.0%) | 1.26 (0.76 to 2.09) | 0.37 |
| 6 months | 14 (7.7%) | 10 (5.9%) | 1.33 (0.58 to 3.09) | 0.50 |
| Parasite density^1^ |  |  |  |  |
| 14 days | 112 (56 to 7895) | 460 (120 to 5800) | -0.44 (-1.64 to 0.77) | 0.47 |
| 6 months | 108 (64 to 540) | 256 (88 to 790) | -0.21 (-1.55 to 1.12) | 0.74 |
| Gametocytemia |  |  |  |  |
| 14 days | 38 (17.5%) | 29 (14.5%) | 1.25 (0.74 to 2.12) | 0.40 |
| 6 months | 14 (7.7%) | 9 (5.3%) | 1.49 (0.63 to 3.54) | 0.37 |
| Parasitemia plus fever^2^ |  |  |  |  |
| 14 days | 6 (2.8%) | 3 (1.5%) | 1.87 (0.46 to 7.57) | 0.38 |
| 6 months | 3 (1.6%) | 1 (0.6%) | 2.83 (0.29 to 27.5) | 0.37 |

Abbreviations: IQR, interquartile range; CI, confidence interval; ^1^Among children with a positive smear, with models using a log transformation of parasite density; ^2^Fever defined as tympanic temperature ≥37.5°C
