# Supplementary material for: Effect of a single dose of oral azithromycin on malaria parasitaemia in children: a randomized controlled trial
Source: Malar J. 2021 Aug 31;20:360. doi: 10.1186/s12936-021-03895-9 (PMC8407066; doi:10.1186/s12936-021-03895-9)
Supplement: Supplementary file 2 — Additional file 2. Height stick for height-based dosing for children aged 12-59 months, Burkina Faso. [file 12936_2021_3895_MOESM2_ESM.pdf]

Zithromax® Pole
for Burkina Faso

FRANCIS I. PROCTOR FOUNDATION
FOR RESEARCH IN OPHTHALMOLOGY
UNIVERSITY OF CALIFORNIA SAN FRANCISCO

- 25ml -

137.7cm

- 19ml -

130cm

- 16ml -

122.2cm

- 14ml -

110.2cm

- 12ml -

98.3cm

- 10ml -

87.5cm

- 8ml -

76.5cm

- 6ml -

65.5cm

- 4ml -

53.8cm

- 2ml -

50.6cm

La toise doit être placée verticalement.
La personne doit se tenir contre la toise
pour calculer la quantité de traitement
(en ml) correspondant à sa taille.

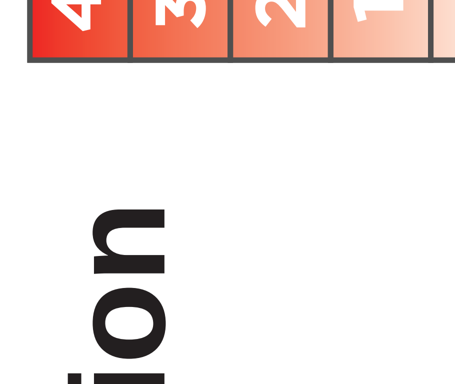

Pour la distribution de Zithromax® aux enfants
